# Developing a population wide cost estimating framework and methods for technological intervention enabling ageing in place: An Australian case

**DOI:** 10.1371/journal.pone.0218448

**Published:** 2019-06-26

**Authors:** Azad Rahman, Delwar Akbar, John Rolfe, Julie Nguyen

**Affiliations:** 1 School of Business and Law, Central Queensland University, Rockhampton, QLD, Australia; 2 School of Health, Medical & Applied Sciences, Central Queensland University, Rockhampton, QLD, Australia; Universidad de Cantabria, SPAIN

## Abstract

**Purpose:**

Ageing in place is one of the greatest desires of elderly people. Assistive digital technologies could potentially delay the institutionalization of the elderly people and allow them ageing in place. This study develops a population-wide cost estimating framework for adopting digital technologies that can improve the quality of life of elderly people through examining an Australian region.

**Methods:**

We developed a five-stage cost estimation framework, which involved progressive forecasting of elderly population and direct cost estimation methods. The forecasting and cost estimation models have been set for a 10-year period because the prediction accuracy from cross-sectional data is better in the short to medium term compared to the long-term. For cost estimation, we categorised the ageing population on the basis of the number of chronic diseases that they have contracted. Costs of assistive technologies were collected from open sources. The model has been tested in the Fitzroy and Central West, a regional area of Queensland in Australia. A stakeholder panel discussion in a workshop format was used to validate the appropriateness of the proposed framework and the study findings.

**Results:**

This study identified eight common chronic diseases with different comorbidity patterns in Australia. We also identified the required assistive technologies to assist patients with chronic diseases. This study estimated that annual per capita cost for technological intervention could range from AUD 4,169 to AUD 7,551 on the basis of different price margins of the technologies.

**Conclusion:**

The approach of categorising the aged cohorts on the basis of the number of chronic diseases helps estimate population-wide costs compared to using single technology intervention costs for a particular chronic disease cohort. The cost estimation framework and the method developed in this study can assist the government to estimate costs for ageing-in-place programs.

## Introduction

One of the most significant social transformations in recent times is the population ageing of the world. Population ageing implies an increase in the share of older persons in the entire population. United Nations (UN) projected that the world population of older persons over 60 years of age will be about 1.4 billion in 2030 with a growth percentage of 56% from the year 2015 [1]. Australia is following a similar trend with increasing numbers of older people. In 2016 approximately 3.7 million people are older Australians (15% of the total population) and by 2031 the number will be between 5.7 to 5.8 million [2].

Ageing in place is one of the most common desires expressed by older Australians [3, 4]. Ageing in place can be defined as the ability to live in one’s own home safely, comfortably and with some level of independence regardless of age and income or ability level [5,6]. However, the presence of chronic disease among the aged cohort may force them to be institutionalised instead of living in their own home. A recent study [7] indicated that more than 11 million Australians suffered from at least one of the eight most common chronic diseases. Meanwhile, about 60% of elderly Australian (aged 65 years and over) have two or more chronic diseases. The comorbidity of the elderly people is the most common reason for shifting them from their own home to care facilities. To enable ageing in place, some studies suggested the adoption of digital technologies for elderly people suffering with or without chronic diseases [8–10].

Technological innovation and adoption to improve the health of elderly people have been improving rapidly since the beginning of the 21st century, especially in the six domains of aged care services: communication technology, technology to support therapy and rehabilitation, telecare and environmental sensors, telehealth, telemedicine and technologies for security. Australian government initiatives and aged care industries have acknowledged the need for technological innovation and adoption in the aged care system [11, 12]. Different technologies are required for different people and purposes. However, to date, no study has been done at a population level to estimate the cost of different technologies that can support the overall health and wellbeing of elderly people.

Cost-benefit analyses on adopting different technologies are available in the literature but not considering the entire population or considering a set of chronic diseases and comorbidity. Some studies investigated both cost and benefits but only a single technology and/or with a single chronic disease. For instance, Kulvik et al. [13] developed an economic model to assess the cost and benefits of applying boron neutron capture therapy (BNCT) to cancer patients. Hoof et al. [14] investigated the ambient intelligence Unattended Autonomous Surveillance system (UAS) and their impact on the lifestyle of the older people living in their own home. However, Hoof et al. [14] did not consider the fact that UAS could be drastically different for an elderly person who is suffering from more than one chronic disease. Hoof et al. [14] concluded that, although the new technologies could increase the sense of safety and security among the sample of elderly people, the ambient intelligence technology alone is not sufficient to enable ageing-in-place. Other forms of technologies like robot technologies [15], smart homes [16], information and communication technologies (ICT) [17], mobile and wearable technologies [8, 18–20] have also been reviewed but only for a single chronic disease and not for different comorbidities.

Akiyama and Abraham [21] provided a comparative cost benefit analysis (CBA) of tele-homecare for two samples of elderly people in Japan with and without government funding support. Caley & Sidhu [22] have also considered a sample group of ageing population in the UK to investigate the health care expenses. They noted that the estimated health care costs could be under or over-estimated due to the uncertainty of the morbidity pattern and the difficulties of measuring the cost burden towards the end of the life. Kok et al. [23] compared the costs and benefits for two samples of elderly people living in home care and residential care. Due to the complex nature of the cost estimation, the researchers deliberately considered only a single technology and/or only one chronic disease. Therefore, a robust framework to estimate the cost of various technologies associated with different comorbidity patterns is missing in the literature. This study aims at filling that gap with an effective cost framework dealing with the entire ageing population of a case study area.

### Background and case study

In the Australian context, no study has been identified as providing a cost analysis of adopting digital assistive technologies for the elderly population. Khosravi and Ghapanchi [24] have conducted a systematic review of the effectiveness of technologies to assist seniors and they noted the shortage of studies conducted on the ageing population of Australia. The health and age care system of Australia is complex due to different types of service providers and funding mechanisms. Currently services and care provided to the elderly population of Australia are delivered in both residential and community based settings. Community based aged care can be categorised in two groups: home care package program and basic home support. Basic home support includes the Commonwealth home support program and the home and community care. To obtain a government supported age care service, elderly people need to be evaluated through an aged care assessment program. Australian government expenditure for aged care is expected to reach $19.8 billion for the year 2018–19. The majority of this expenditure is to support residential care (66.2%) followed by home support (16.9%) and home care (11.6%) [25]. Based on the assistance required and overall circumstances of the aged persons needing help, the assessment team refers them to potential service providers. According to ACFA [25], there are 2,376 service providers for aged care in Australia. Most service providers offer a single type of age care service, but some offer two or all three types of services.

**Fig 1:**
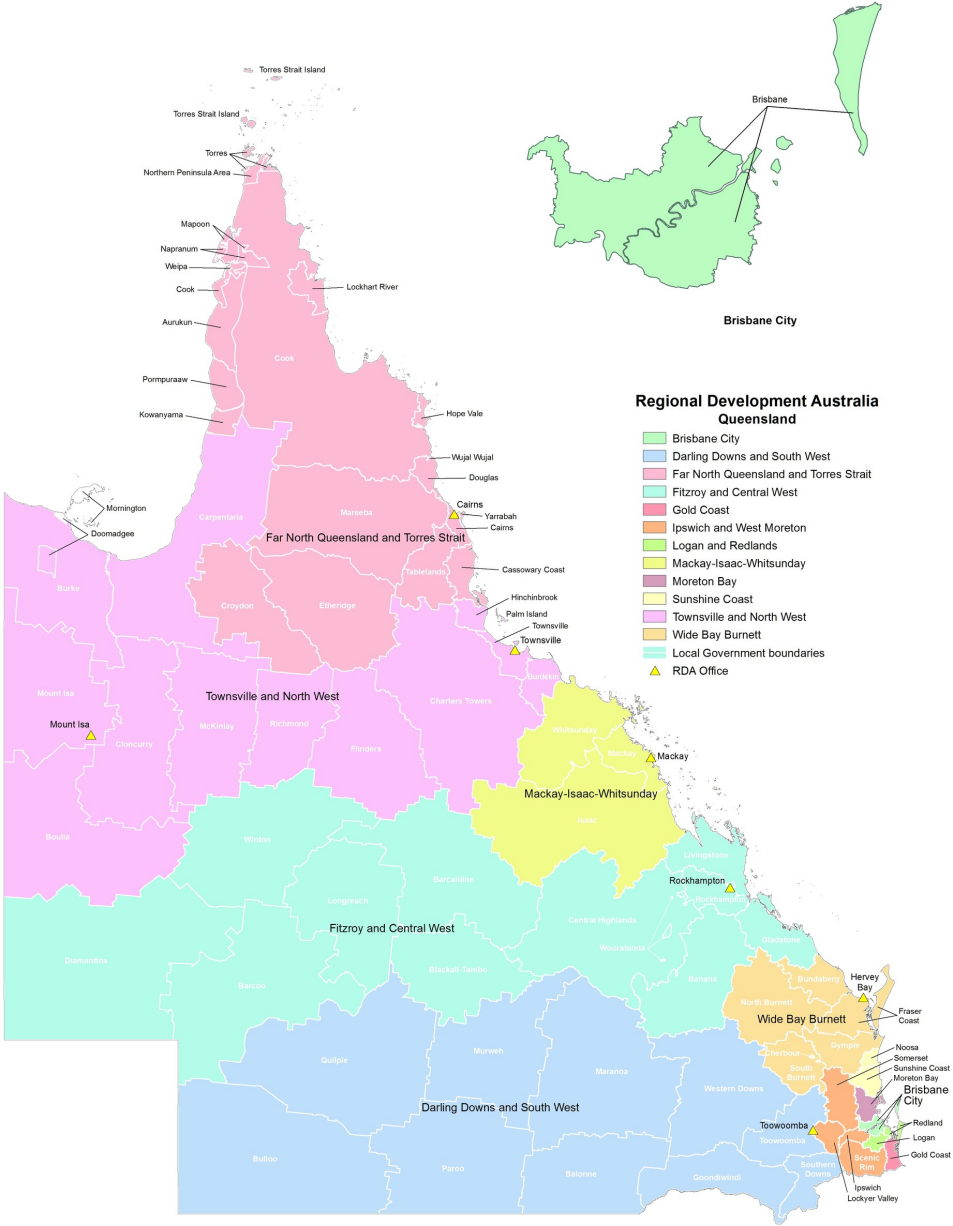
Location of FCW region in Queensland (Reprinted from Commonwealth of Australia Queensland RDA [27] under a CC BY license, with permission from RDA, original copyright 2019).

The Fitzroy and Central West (FCW) (Fig 1) is one of the largest regions of Queensland, Australia, with a total land area of 452,454.2 km^2^, approximately 26% of Queensland [26]. The estimated resident population of FCW was 236,134 people on 30th June 2017 [26].

In 2016 13% of FCW region residents were aged over 65, which is very similar to the state of Queensland and the national level ageing population (Table 1). A summary of the population data is presented in Table 1. Recent data indicate that currently there are 76 aged care service providers in the FCW region which provide different types of care including residential care, home care and transition care [28].

**Table 1:** Estimated resident population of FCW region, by age group at 30 June 2016.

Age (years)	Estimated Population [29]					
	Australia	% of Total Australian population	Queensland	% of Total Queensland’s population	FCW	% of FCW
65–69	1,194,248	4.93	241,437	4.98	10410	4.40
70–74	890,221	3.68	179,601	3.70	7536	3.19
75–79	651,134	2.69	125,049	2.58	5495	2.32
80–84	455,177	1.88	82,629	1.70	3615	1.53
85 and over	482,731	1.99	84,937	1.75	3258	1.38
**All ages (total)**	**24,210,809**		**4,848,877**		**236,599**	

Source: Australian Demographic Statistics, available from: http://www.ausstats.abs.gov.au/ausstats/subscriber.nsf/0/8B46E5DBE0FC9549CA2582570013F721/$File/31010_sep%202017.pdf

This study considers the total ageing population of the FCW region and their comorbidity patterns on the basis of available literature. Then appropriate digital technologies to assist elderly people who like to stay at their own home within this region are identified.

### Elderly people need assistance for ageing in place

Among Australian elderly residents, about 39% need assistance with their day to day activities. Assistance might be required for activities like self-care, mobility, communication, cognitive or emotional tasks, health care, reading or writing tasks, transport, household chores, property maintenance, and meal preparation. The data indicates that 94.8% of elderly people are living in households in contrast to 5.2% who are living in cared-accommodation [30]. The summary of findings is included in Table 2.

**Table 2:** Percentage of Australian population aged 65 years and over living in their own homes and requiring assistance.

	Age group (years)						
	65–69	70–74	75–79	80–84	85–89	90 and over	Total
All needing assistance with at least one activity	22.2%	29.1%	39.4%	56.8%	72.5%	88.5%	38.6%
Assistance not needed	77.8%	71.0%	60.6%	43.0%	27.5%	11.8%	61.4%
Living in cared-accommodation	0.8%	1.4%	3.2%	7.7%	17.0%	37.0%	5.2%
Living in households	99.2%	98.6%	96.8%	92.3%	83.0%	63.0%	94.8%
Living in households and need assistance	22.03%	28.72%	38.20%	52.43%	60.18%	55.78%	**36.61%**
**Total (in thousands)**	**1,149.7**	**859.6**	**630.2**	**444.0**	**297.8**	**165.7**	**3,546.2**

Source: Disability, Ageing and Carers, Australia [30], available from: http://www.abs.gov.au/ausstats/subscriber.nsf/log?openagent&qld_2015.xls&4430.0&Data%20Cubes&E5B39185DA86339DCA2580A500115EAF&0&2015&12.01.2017&Latest.

The results of the combinatorial analysis indicate 36.61% of the Australian elderly population require assistance with at least one activity while they are living in their own home (Table 2).

### Number of diseases of people aged 65 years and over

Many Australians aged over 65 are suffering one or more of eight most common chronic diseases. Table 3 indicates the number of Australians aged 65 years and over with one or more chronic diseases. Among the cohort, 13.6% don’t have any chronic disease but they might need some basic technologies to assist them; 26.6% have only one primary chronic diseases; 30.6% have two chronic diseases while another 29.3% have three or more chronic diseases [31]. People with chronic diseases require assistive technologies, and additional technological assistance may be required for elderly people who have 2 or more chronic diseases.

**Table 3:** Number of chronic diseases of Australian population aged 65 years and over (in 1000).

	Number of Chronic Diseases (in 1000).				
Chronic Diseases	0	1	2	3 or more	Total
Arthritis	. .	237.8	653.4	772.9	1,665.1
Asthma	. .	28.4	69.9	236.1	339.7
Back problems (dorsopathies)	. .	95.6	204.7	550.0	853.7
Cancer (malignant neoplasms)	. .	15.9	53.1	121.8	191.9
Chronic obstructive pulmonary disease (COPD)	. .	8.9	37.8	216.7	257.5
Diabetes mellitus	. .	57.3	146.5	373.1	573.0
Diseases of the circulatory system / Cardiovascular disease (CVD)	. .	389.1	701.6	834.9	1,920.5
Mental and behavioural problems	. .	43.0	134.1	402.4	584.4
Total persons aged 65 years and over (in 1000)	**447.2**	**872.6**	**1,004.3**	**962.4**	**3,285.6**
Percentage	**13.6%**	**26.6%**	**30.6%**	**29.3%**	

Source: National Health Survey, available from: http://www.ausstats.abs.gov.au/ausstats/subscriber.nsf/0/CDA852A349B4CEE6CA257F150009FC53/$File/national%20health%20survey%20first%20results,%202014-15.pdf.

This study employs the commodity patterns of Australian ageing population classified by the national health survey [31]. The following Table 4 illustrates the comorbidity matrix for the selected eight chronic diseases. Table 4 indicates that the most common comorbidities are Arthritis and CVD; Arthritis and Back pain; and CVD and Back pain [31].

**Table 4:** Comorbidity matrix of chronic diseases in the Australian population aged 65 years and over (in 1000).

Primary chronic disease	Arthritis	Asthma	Back problems	Cancer	COPD	Diabetes	CVD	Mental and behavioural problems
Arthritis	. .	202.5	553.6	89.8	174.5	299.1	1,059.1	368.0
Asthma	202.5	. .	126.8	23.8	91.9	80.3	215.2	82.1
Back problems	553.6	126.8	. .	55.3	106.0	184.3	533.3	209.9
Cancer	89.8	23.8	55.3	. .	18.2	39.5	128.6	54.2
COPD	174.5	91.9	106.0	18.2	. .	62.3	188.7	84.8
Diabetes	299.1	80.3	184.3	39.5	62.3	. .	421.6	131.7
CVD	1,059.1	215.2	533.3	128.6	188.7	421.6	. .	378.1
Mental and behavioural problems	368.0	82.1	209.9	54.2	84.8	131.7	378.1	. .
**Total persons aged 65 years and over**	**1,665.1**	**339.7**	**853.7**	**191.9**	**257.5**	**573.0**	**1,920.5**	**584.4**

Source: National Health Survey, available from: http://www.ausstats.abs.gov.au/ausstats/subscriber.nsf/0/CDA852A349B4CEE6CA257F150009FC53/$File/national%20health%20survey%20first%20results,%202014-15.pdf.

## Methodology

This section provides a detailed description of the methodology applied to collect data and the quantitative approach used to assess and analyse associated costs. Secondary data on the ageing population and the expenditure in the allied health sector were collected from Australian Bureau of Statistics (ABS) [32], Australian Institute of Health and Welfare (AIHW) [33] and Aged Care Financing Authority (ACFA) [34]. Suitable assistive digital technologies have been identified from the available literature, medical catalogues, online resources and current usage by the health practitioners and service providers. The price of the identified technologies was mostly collected from online sources and by sourcing quotes from retailers and suppliers.

### Identification of relevant technologies, costs and total cost estimation

A five step hybrid model has been developed in this paper that includes progressive forecasting and direct cost estimation methods to estimate the total costs of adopting digital technologies for the elderly population. The population data of elderly people within the case study region FCW are available from a government website including information about their residential status. In the first step, a progressive forecasting method was used to predict the aged population in the FCW region over the next 10-year period, taking account of both inflows (ageing cohorts and in-migration) and outflows (deaths and out-migration). The death rates of specific age groups and migration rates for both overseas and interstate populations are estimated through a linear regression model drawing on historic data from government databases. For estimating death rates, the population of QLD and FCW, year and the number of people with one or more chronic diseases in the different age groups were used as independent variables, while for migration (both interstate and overseas), only the population of QLD and FCW and year were used as independent variables.

In the second step, this study investigated the comorbidity pattern of elderly people living in the FCW region grouped into four categories based on the number of chronic diseases they have. Since individual data are not available due to confidentiality restrictions, the number of people for each category with possible combinations of chronic diseases were estimated using normalisation ratios and combinatorial methods. This prediction is vital for cost estimation as the different categories of elderly people need different sets of technologies, each with specific costs.

In the third step all direct costs related to technology adoption such as initial purchasing, set-up and maintenance costs have been estimated based on the prices available from open access online sources. Then a literature review was conducted to understand the needs for the technologies that suit adoption by elderly people who choose to live in their own home. In the final step a cost estimation model considered all possible comorbidities pattern (more than one chronic disease) and calculated the cost for each possible combination. The details of the calculation procedure are described in the cost estimation section.

**Fig 2:**
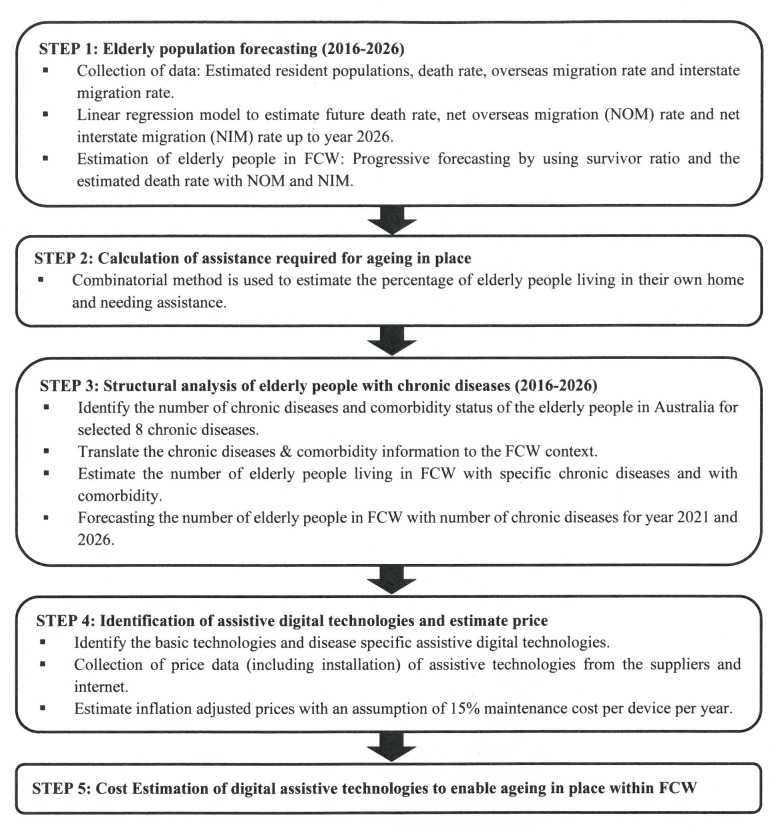
Framework for cost estimation.

The forecasting and cost estimation models have been set for a 10-year period because many government planning and forecasting models (e.g. Queensland population predictions) use 10 year horizons. While accuracy of model predictions is likely to be higher with shorter horizons, predictions over the medium term (e.g. ten years) is more likely to be helpful for planning policy development. Longer time frames are unlikely to be realistic because technologies are changing very rapidly and costs could be very different after 10 years. Total costs have been estimated based on eight key chronic diseases of the FCW population aged over 65 years and over. All costs (fixed and variable costs) are estimated in the 2016 base year value. The results presented indicate the average cost required for the assistive technologies to enable ageing in place. The five steps in the hybrid model for cost estimation are presented in Fig 2.

**Fig 3:**
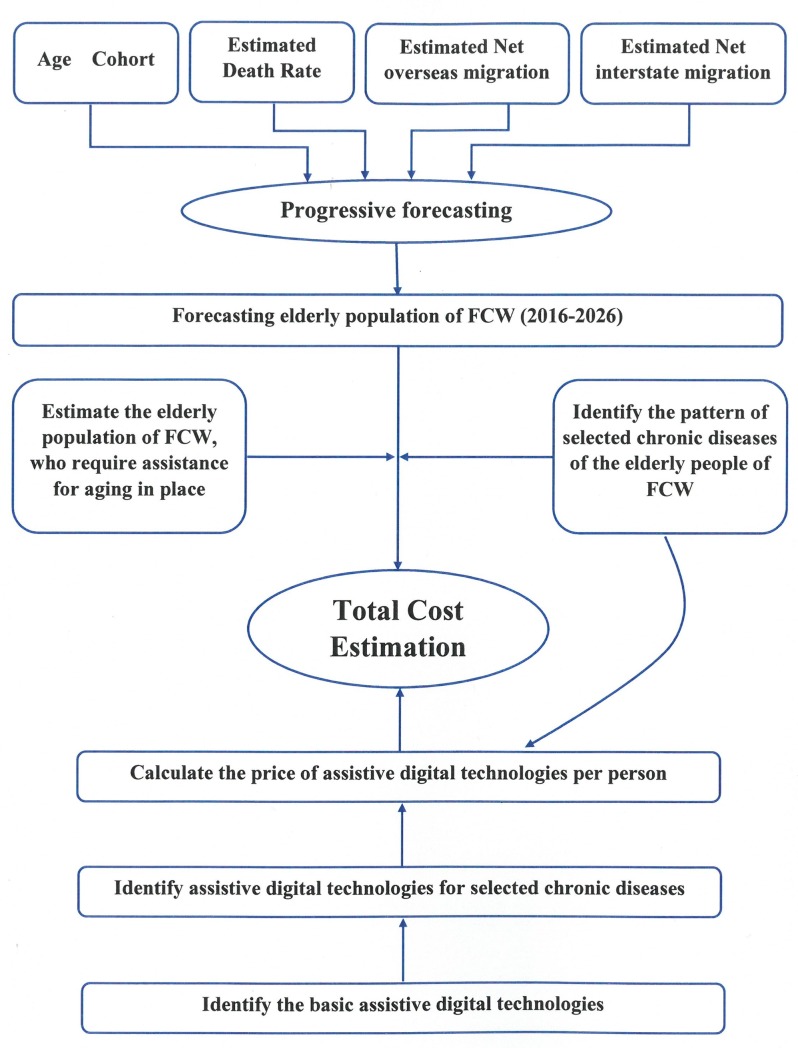
Operational model for cost estimation for technological adoption.

The operational model with the data flow direction is presented in Fig 3. The model has been built in a Microsoft Excel file with multiple worksheets. Worksheets are connected by formulas and it is a macro-enabled workbook.

### Cost analysis result validation workshop

A stakeholder panel (such as key reference or representative group) is an appropriate approach to validate any resource/cost management framework as well as reaching an agreement about suitable digital technologies for a particular group such as for an elderly population. This method (i.e., stakeholder panel discussion) has been used in the past far validating costs estimation for resource planning [35] and for telemedicine application [36]. This study used a stakeholder panel discussion in a workshop format to validate the appropriateness of the proposed framework, digital technologies and cost findings. In order to form a stakeholder panel, representative stakeholders from state and federal governments, health practitioners, aged care assessment teams from FCW region and academic experts were engaged. The workshop format and processes were approved by the CQUniversity Human Research Ethics Committee (Approval no. 21152). The research team initially contacted 22 stakeholders, with 18 of them consenting to participate and 13 of them attending the workshop. The participants included four were directly providing health care support to the elderly people, two practitioners/ therapists, two federal government officers, one social worker from central Queensland health service, one IT specialist from service providers, and three researchers. The diversity and experience of the stakeholders involved helped to validate the cost estimation model. The four key discussion points emerging from the stakeholders’ workshop were cost estimation frameworks, technologies considered for the estimation, comorbidity patterns and priorities for future study. The stakeholder panel acknowledged the appropriateness of the cost estimation framework with some minor adjustments, which were subsequently incorporated into the model. The stakeholders added several digital technologies to the list of the technologies available in the literature for the elderly population with one or more than one chronic diseases. The stakeholders were aware of the changing nature of the comorbidity patterns but they endorsed the use of the national comorbidity pattern published by the Australian government.

### Identification of comorbidity and assistive technologies

Different illness and health conditions are frequently referred to as a chronic disease. According to AIHW [37], chronic diseases are long-lasting conditions with persistent effects. Numerous generalized definitions of chronic diseases are available in the literature; however, a standard definition is not available. National Health Survey (NHS) [31] reported that most of the elderly people (age over 65) are suffering from one or more of the following eight chronic diseases: Arthritis, Asthma, Back pain and problems, Cancer (such as lung and colorectal cancer), Cardiovascular disease (CVD), Chronic obstructive pulmonary disease (COPD), Diabetes, and Mental health conditions. Types of care and assistive technologies needed by a consumer differ from person to person based on the number of chronic diseases he/she has contracted. However, the standard of basic care (not technologies) of elderly people mostly depends on the number of chronic diseases, as presented in Table 5.

**Table 5:** Care needs categories.

Category	Number of Chronic diseases	Needs
Basic	Nil chronic diseases	■ Basic
Low Care	At least one of the eight selected chronic diseases	■ Lives alone or with family/carer. Needs fortnightly cleaning and community access
Intermediate Care	Two or more of the eight selected chronic diseases	■ Lives alone or with family/carer ■ Cleaning and community access ■ Requires medication monitoring and/or administration of medication ■ Mobility difficulties ■ Activity daily living difficulties ■ Increase of fall risk
High Care	Three or more chronic diseases	■ As above (Though the needs of this cohort are same as the previous one, they may need additional number of assistive technologies depending on their comorbidity pattern.)

According to the national health survey [31], about 14% of elderly people living in Australia do not have any chronic diseases. However, this cohort may have other non-chronic issues like vision impairment, hearing problems, communication difficulties and fitness issues requiring some basic technologies to support their ageing in place, so they are categorised in the basic care level category. The second category is comprised of elderly people with single chronic diseases, with 26.6% of elderly people of Australia falling into this category. The next two categories are comprised of elderly people with two chronic diseases and three or more chronic diseases respectively. Table 5 illustrates the support may be needed by the different categories of people aged over 65 with no chronic disease or some chronic diseases.

The concept of assistive technologies refers to the process of integrating technologies within the residence to achieve improved functional health, safety, security and quality of life [38]. A wide range of assistive technologies are currently available and being used by elderly people in Australia. To date, no extensive research has been conducted to identify the types of technologies for different cohorts of elderly people. Identifying appropriate technologies is vital for predicting the services required for various groups of elderly people. Technologies can be categorised on different aspects including functionality, price and usage. The most common functions of the assistive technologies are providing aid for mobility, vision, hearing, environmental/home safety, exercise and fitness, health monitoring and cognition. The health monitoring devices are designed for cohorts with specific chronic diseases. For example, the ECG self-monitoring device and blood pressure monitors are two important devices for patients with cardiovascular diseases. On the other hand, some technologies could be used by elderly people irrespective of their chronic diseases, such as mobility devices (e.g., electric motorised wheelchair), home safety devices (e.g., fall detectors/motion sensors, exercise and fitness devices (e.g., treadmill and exercise bike). In the current cost estimation framework, technologies are categorised based on the needs of elderly people who are suffering from different chronic diseases, with other non-specific technologies excluded from the analysis. A summary of potential digital assistive technologies for the elderly people that has been identified through an extensive literature review is provided in S1 Appendix.

The costs of the technologies vary from company to company because of variations in the functionality, reliability and servicing costs. The cost data for the technologies were collected mostly from online sources, medical catalogues and by personal communication. A complete list of the technologies with price and source of the price is given in S2 Appendix. Because of the wide range of prices, this study considered two scenarios with the lowest price and average price of each technology. In addition, an assumption was made that the life span of these technologies would be five years and after which the devices would need to be replaced by new sets.

Maintenance costs of the technologies also vary from device to device and company to company, with most medical equipment requiring maintenance at least once a year (sometimes even more frequently) to ensure the performance and the reliability of the equipment. While some Australian states have guidelines for the management of medical equipment in government hospitals [39, 40], management guidelines for the same equipment for household uses are not available. A report published by the Auditor General of Victoria [39] identified that the maintenance costs of these types of equipment in public hospitals varied from 1% to 12%, while the Auditor General of Western Australia [40] indicated that in the year 2015–16, about 22% of the total medical equipment expenditure was spent on the maintenance and repairs. Equipment installed in home settings may require more maintenance since the equipment will be handled by non-professionals, as well as higher installation costs. Based on the available information a 15% maintenance cost for each technology per annum was assumed. An installation cost was also included and assumed to be 5% of the device’s price.

## Results and findings

### Elderly population forecasting for RDA FCW region

A progressive forecasting method with a survivor ratio and estimated death rate, was used to estimate the ageing population of FCW for the next 10 years. Initially, the estimated population and forecasted death rates of elderly aged group people are determined by using a regression model. Net overseas migration (NOM) and net interstate migration (NIM) rate were used to calculate future trends (NOM rate = −0.006% and NIM rate = 0.058%). The population data from 2006, 2011 and 2016 censuses were used to estimate population growth and death rates. The results are summarised in Table 6. The results indicate that the total population of FCW will decrease by 7% by the year 2026 while the population for Queensland will increase by 20%. The death rates of the 65+ age groups are likely to decrease for the next 10 years.

**Table 6:** Death rate–Qld (Green boxes indicate the estimated results).

Year	2006	2011	2016	2021	2026	Changes from 2016 to 2026
Population QLD	4007992	4476778	4848877	5191483	5835658	20.3%
Population FCW	210637	229056	236599	235440	220131	- 6.9%
Age Group	Death Rate					
55–59	4.40	4.20	4.10	4.02	3.69	-10.0%
60–64	7.00	6.60	6.00	5.42	4.84	-19.3%
65–69	11.80	10.30	9.60	8.54	6.38	-33.5%
70–74	19.00	17.70	15.50	12.30	12.17	-21.5%
75–79	33.10	29.50	27.10	24.54	20.20	-25.5%
80–84	59.70	54.90	49.80	42.96	40.55	-18.6%
85–89	104.00	103.90	94.50	84.46	85.83	-9.2%
90–94	186.90	183.40	167.50	154.74	154.88	-7.5%
NOM for FCW				-14	-13	
NIM for FCW				137	128	

NOM and NIM are also considered during the estimation, and the results are summarised in Table 7. According to the estimated results, 20% of the population will be aged over 65 by 2026 compared to 13% in 2016 (Table 7). The total increase between 2016 and 2026 in the number of people over 65 years of age will be 44.5%.

**Table 7:** Progressive forecasting on elderly population of FCW (Green boxes indicate the estimated results).

Age Group	Year				
	2006	2011	2016	2021	2026
55–59	12154	14281	15432		
60–64	9481	11703	12475	15014	
65–69	7371	8720	10410	11967	14558
70–74	5645	6387	7536	9794	11262
75–79	4527	4762	5495	6636	8828
80–84	2948	3432	3615	4339	5313
85+	2349	2698	3258	3423	3863
total 65+	22840	25999	30314	36159	43824
Total	210637	229056	236599	235440	220131
%	11%	11%	13%	15%	20%

The estimated results for the elderly population in FCW with no to multiple chronic diseases in 2016, 2021 and 2026 are presented in Table 8. The number of individuals in FCW with specific chronic diseases and with different comorbidities has also been determined for cost estimation purposes.

**Table 8:** Estimated population of FCW with different numbers of chronic diseases.

Year	2016	2021	2026	%	Ref
Estimated Population of FCW	30314	36159	43824		Table 7
Living in household and need assistance	11098	13238	16044	36.61%	Table 2
no chronic diseases	1511	1802	2184	13.6%	Table 3
One primary chronic disease only	2947	3516	4261	26.6%	Table 3
Two chronic diseases	3392	4046	4904	30.6%	Table 3
Three or more chronic diseases	3251	3878	4699	29.3%	Table 3

### Costs estimation

The cost estimation task is carried out in two phases. In the first stage the price of assistive technologies and the number of elderly people with one chronic disease were identified and estimated (Table 9). Though the general comorbidity patterns of the Australian ageing population are accessible, the individual data for the elderly people with their comorbidity patterns were unavailable. We used the available data and some assumptions to calculate the costs.

**Table 9:** Breakdown of FCW elderly population with one chronic disease.

Year	2016	2021	2026	
FCW population with one chronic disease	2947	3516	4261	% (Ref Table 3)
Arthritis	805	960	1163	27.3%
Asthma	97	116	141	3.3%
Back problems (dorsopathies)	324	387	469	11.0%
Cancer (malignant neoplasms)	53	63	77	1.8%
Chronic obstructive pulmonary disease (COPD)	29	35	43	1.0%
Diabetes mellitus	195	232	281	6.6%
Diseases of the circulatory system	1314	1568	1900	44.6%
Mental and behavioural problems	144	172	209	4.9%

The total costs for the assistive technologies are calculated by multiplying the size of the estimated population with one chronic disease with the costs associated with their potential need based on their primary disease. For the population with two chronic diseases, some overlapped (the same individual is counted in different categories) data are available in the ABS databank (Table 4). Most common combinations of diseases are Arthritis & CVD; Arthritis & Back Problem and CVD & Back Problem. There are 28 possible combinations for the eight most common diseases. For cost calculations, all combinations were considered and the percentage of the population in each was determined by using normalized ratios.

Similar scenarios were considered for the population with three or more chronic diseases. Most common combinations of diseases are:

- Arthritis, CVD and back problem
- Arthritis, CVD and mental health
- Arthritis, CVD and diabetes
- CVD, mental health and back problem

Some assumptions were made to facilitate the cost estimation:

- 219 possible combinations were considered in this study for three or more common diseases among the selected eight diseases.
- For cost calculations, the most common combinations were considered and population percentages were estimated using combinatorial methods.
- The rest were calculated by using weighted average methods.

The costs estimation results are presented in Table 10. The price data were collected in 2018 while the population data were from the 2016 census. Therefore, we adjusted the 2018 prices of technologies to 2016 prices (Table 10) by using the average inflation rate between 2016 and 2018. The 2016 prices are used as the base price for all costs in the study. As the price of assistive technologies was highly variable (see S2 Appendix), estimates of both low and average costs are used in the analysis.

**Table 10:** Cost estimation for assistive technologies.

	Cost unit	2016		2021		2026	
		low	Average	low	Average	low	Average
No chronic diseases	AUD	3,458,063	7,864,246	4,124,789	9,380,500	4,999,183	11,369,026
One primary chronic disease only	AUD	11,508,369	20,045,598	13,730,378	23,915,956	16,639,688	28,983,473
Two chronic disease	AUD	15,052,611	27,092,848	17,954,805	32,316,440	21,760,956	39,167,043
Three or more chronic disease	AUD	16,244,548	28,795,217	19,376,552	34,347,032	23,484,092	41,628,090
**Total**	AUD	**46,263,591**	**83,797,909**	**55,186,525**	**99,959,928**	**66,883,918**	**121,147,631**

**Fig 4:**
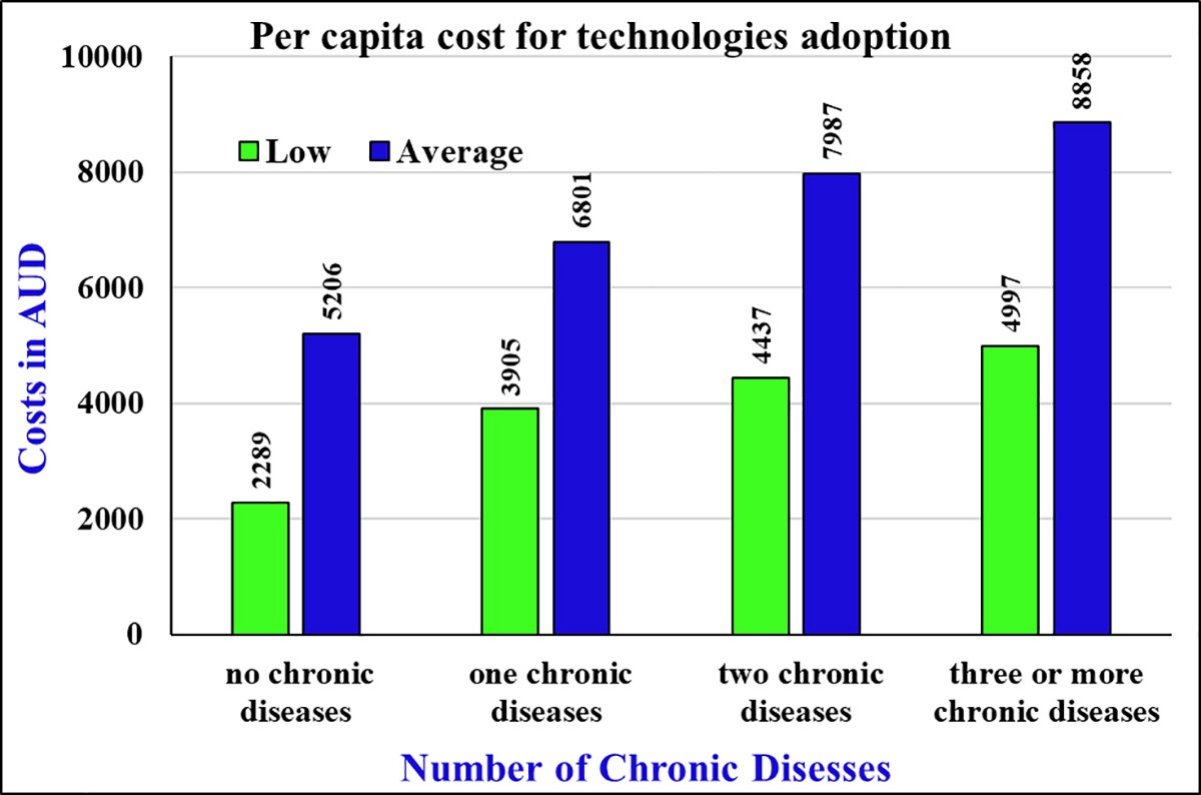
Per capita cost for technologies adoption for the different groups.

The results indicate that per annum and per capita costs for the 10-year period could be AUD 4,169 for the low cost scenario and AUD 7,551 for the average one. Due to the increasing elderly population in FCW, total annual cost could increase from AUD 46.2 million to AUD 66.8 million (low cost case) respectively from the year 2016 to the year 2026 (Table 10). Per capita costs vary amongst the elderly people by the number of chronic diseases that an individual has. Fig 4 illustrates the variation in per capita costs of adopting assistive technologies with different groups of people.

## Discussion

In this study, we have developed a framework to estimate the costs of technological intervention to enabling ageing in place. The key findings of the current study are the annual per capita cost for the technologies and total net costs for a 10-year period. The results indicate that the costs are dependent on two determinants; the first is the number of chronic diseases contracted by the individuals and the second is the price of the digital assistive technologies. The annual per capita cost could be more than double for an elderly person with three or more chronic diseases compared to no chronic diseases. This study measured total costs with low and average prices of the technologies, with results indicating that the difference of annual per capita costs could be about AUD 3,800 for an elderly person with multiple chronic diseases.

An important contribution of this paper is the cost estimation framework and the methods used to apply this framework to a population level. The cost estimation framework is designed to estimate the cost in five different stages over a 10-year period A wide range of technologies was taken into account for the cost estimation and categorized on the basis of their functionality to assist elderly people with or without chronic diseases. This approach is justifiable as the needs for assistive digital technologies are different for the ageing cohorts with comorbidity.

Akiyama and Abraham [21] reported that two components need to be considered during the cost estimation of technological intervention, namely, the initial cost, which includes system integration and the device cost; and operational cost. In the current study, we have included both components for the suggested time frame. Akiyama and Abraham [21] studied two different models to identify the cost of a tele home care system for elderly people suffering from chronic diseases. However, in their estimation, the devices considered were only to support the tele home care system. Unlike the approach of Akiyama and Abraham [21], we include all possible digital devices for assisting elderly people living at home. Dang et al. [41] have also investigated the usage of tele home care but focused on elderly people with heart diseases. In the current study, eight chronic diseases are included for analysis and hence a wide range of devices are categorised to cover the support needed for elderly people with one or more chronic diseases.

One of the key aspects of the framework employed in this study is the estimation of the number of elderly people using progressive forecasting methods. In the progressive forecasting technique, the survivor ratio of the population of an age group determines their entry to the next age group in the coming years. For a medium term analysis (10 years for our current study) this strategy is reasonable as the birth rate of the population did not affect the numbers in older cohorts. For greater accuracy, we also consider the net overseas and interstate migration for the selected age group (65 years and over). The current cost estimation is performed for the entire elderly population of FCW region, which is another novel approach. Most of the cost estimations previously done have been conducted for a small sample group with single technology intervention. For instance, Magnusson & Hanson [17] conducted a cost analysis for information and communication technologies (ICT) adoption by the elderly people in Sweden by selecting only five families for their trial. In contrast, Kok et al. [23] conducted a cost benefit analysis on home care and residential care program by selecting all elderly people of a selected region of Netherlands; they used past survey data and excluded all individuals who did not meet the criteria of the model. Similar to the Kok et al. [23] model, we collected population data from a government data bank (i.e., ABS) and excluded all persons aged under 65 years. The elderly population are then categorised on the basis of the number of chronic diseases and the support they need to stay in their home.

The cost estimation framework considered the comorbidity pattern of the ageing population as it has a major effect on the technologies required and hence on the estimated costs. The structural analysis of elderly people with chronic diseases is a unique approach of our framework as most of the past research only considered a single chronic disease and associated technologies. In the direct cost estimation stage of analysis, the life cycles costs of the assistive technologies are incorporated in the framework to ensure better accuracy.

Australian governments spent approximately $5.3 billion for the year 2017–18 for basic home support and home care [25]. The current cost estimation framework provides an important guideline for the government for utilising their spending in an appropriate way to ensure maximum ageing in place. This cost estimation framework could be more effective for the low socio-economic countries where ageing in place is a common trend due to insufficient care facilities and health professionals.

The current study has some limitations. First, the estimation could differ from person to person on the basis of their choice and adoption capacity of digital technologies. Individual data could be collected through a randomized control trial (RCT), which may facilitate the estimation of benefits on adopting the assistive digital technologies. As primary data was not collected, the benefits analysis was not included in this study. Data collected from the literature did not contain individual information regarding the comorbidity pattern. Hence, some basic assumptions were made during the cost estimation, which may affect the total figure.

Second, this study did not include a detailed benefits analysis of technological interventions. According to the current aged care system of Australia, the cost of technological intervention should be covered by different programs of federal and state governments. Without a proper benefit analysis, such spending could not be justifiable. Finally, the current study is conducted in the Australian context, thus the generalization of the findings internationally could be limited because of the availability of technologies and services can be different in different countries.

## Conclusion

This study has developed a framework and methods to estimate the costs for adopting assistive digital technologies for the wellbeing of an ageing population, which has not been developed before for the Australian elderly population. Then this framework is applied to the FCW region for a 10-year timeframe. First, this study has used the progressive forecasting method to estimate the number of elderly people in the FCW region. The study found that there will be a 45 percent increase in residents in the FCW region aged 65 years and over a 10 year period. Second, the study identifies the comorbidity pattern of the elderly population of the FCW region to assess the range of digital technologies that might be required to assist them at their home.

Costs were estimated based on the comorbidity matrix and relevant technologies required for each group. This study found, for the low-cost device users, the per capita cost will be AUD 4,169 per year while the figure could be as high as AUD 7,551 per year for the digital devices with average range price. For the elderly people with no chronic diseases, per capita costs for technological intervention is predicted to be AUD 2,289 with low priced devices and AUD 5,206 for average-priced devices. The results reveal that comorbidity pattern affects the per capita technological intervention cost. For instance, with the average-priced devices and for the elderly people with three or more chronic diseases, the annual per capita costs could reach to AUD 8,858.

The cost estimation framework developed in this study has potential to be an important tool for service providers and policymakers in Australia. One of the key components of the cost estimation framework is the categorisation of the ageing population centred on the different chronic diseases affecting different cohorts. This categorization allows more precise estimation of the technology and care requirements, as well as the costs involved. While the approach demonstrated in this study has used only eight chronic diseases and a number of simplifying assumptions to estimate rates of incidence, comorbidity and costs, there is potential to develop more sophisticated approaches with more precise data and categorisation.

## Supporting information

S1 Appendix Technologies to support chronic diseases patients. (PDF)

S2 Appendix List of price of digital assistive technologies for elderly people. (PDF)
