# Supplementary material for: Developing a population wide cost estimating framework and methods for technological intervention enabling ageing in place: An Australian case
Source: PLoS One. 2019 Jun 26;14(6):e0218448. doi: 10.1371/journal.pone.0218448 (PMC6594731; doi:10.1371/journal.pone.0218448)
Supplement: S1 Appendix — (PDF) [file pone.0218448.s001.pdf]

S1 Appendix. Technologies to support chronic diseases patients.

<table>
<tr><th colspan="3">Nil Chronic disease/Low care</th></tr>
<tr><td rowspan="6">Function</td><td>Mobility</td><td>▪ Cane/sticks (non-digital) or crutches (non-digital)
▪ Walking frames i.e. 2 wheels, 4 wheels (non-digital)
▪ Chairs for shower/bath/toilet (non-digital)</td></tr>
<tr><td>Vision</td><td>▪ Lighting i.e. lamps, touch lamps
▪ Magnification/Magnifiers (digital and hand-held)
▪ Audio player with DAISY capability Watch (digital)
▪ Big button phone</td></tr>
<tr><td>Hearing</td><td>▪ Alarm signallers with light/sound vibration
▪ Hearing aids (digital) and batteries
▪ Sound amplifier
▪ Amplified phone
▪ Pocket talker
▪ Audio books</td></tr>
<tr><td>Communication</td><td>▪ Communication software
▪ Deaf/blind communicators
▪ Keyboard and mouse emulation software
▪ Personal digital assistant (i.e. iPad)
▪ Video communication devices i.e. phone with screen
▪ Mobile devices- phones/smartphones, Simplified phones
▪ Computers
▪ Large format keyboards</td></tr>
<tr><td>Environment/Home safety</td><td>▪ Power failure alarm with flashlight</td></tr>
<tr><td>Exercise and Fitness (no more than one)</td><td>▪ Pedal exercisers/pedal bike
▪ Treadmill
▪ Exercise bike</td></tr>
<tr><th colspan="3">Particular Chronic disease</th></tr>
<tr><th colspan="3">Arthritis</th></tr>
<tr><td rowspan="3">Function</td><td>Basic needs</td><td>Same as Nil chronic diseases category</td></tr>
<tr><td>Environment/Home safety</td><td>▪ Kitchen tools- food processor
▪ Electric jar/can opener
▪ Electric toothbrush
▪ Robot vacuum</td></tr>
<tr><td>Exercise and fitness</td><td>▪ Exercise bike</td></tr>
<tr><th colspan="3">Asthma</th></tr>
<tr><td rowspan="2">Function</td><td>Basic needs</td><td>Same as Nil chronic diseases category</td></tr>
<tr><td>Health monitoring devices/chronic disease related</td><td>▪ Spirometer
▪ Pulse oximeter
▪ Electronic Asthma monitoring
▪ Nebuliser system
▪ Air purifier</td></tr>
<tr><th colspan="3">Back Pain</th></tr>
<tr><td rowspan="4">Function</td><td>Basic needs</td><td>Same as Nil chronic diseases category</td></tr>
<tr><td>Health monitoring devices/chronic disease related</td><td>▪ Thermal belt
▪ TENS machine</td></tr>
<tr><td>Environment/Home safety</td><td>▪ Hoist/lifter
▪ Robot vacuum</td></tr>
<tr><td>Mobility</td><td>▪ Recliner Chairs</td></tr>
</table>

	Exercise and Fitness	▪ Pedal exercisers/pedal bike
**Cancer**		
Function	Basic needs	Same as Nil chronic diseases category
	Health monitoring devices/chronic disease related	▪ Nebuliser<br>▪ Air purifier
	Cognition	▪ Pill organises and reminder<br>▪ Personal emergency alarm system
	Mobility	▪ Recliner Chairs<br>▪ Electric motorised wheel chair<br>▪ Electric scooter (*assumption- wheelchair or scooter)
	Environment/Home safety	▪ Fall detectors/Motion sensors/Fall monitor<br>▪ Personal emergency alarm system<br>▪ Pressure care mattress (*assumption-may require temporarily)<br>▪ Hospital bed (*may require temporarily)<br>▪ Hoist/lifter<br>▪ Medical Alert system with risk of falls/duress alarm
**Cardiovascular Disease**		
Function	Basic needs	Same as Nil chronic diseases category
	Health monitoring devices/chronic disease related	▪ Blood pressure monitoring<br>▪ INR monitoring tester<br>▪ Heart rate monitor<br>▪ ECG self-monitoring<br>▪ Medication tablet reminder<br>▪ Home monitoring devices for vital signs<br>▪ Thermometer
	Mobility	▪ Recliner Chairs<br>▪ Electric motorised wheel chair<br>▪ Electric scooter (*assumption- wheelchair or scooter)
	Cognition	▪ Pill/Medication organises and reminder<br>▪ Personal emergency alarm system
	Environment/Home safety	▪ Fall detectors/Motion sensors/Fall monitor<br>▪ Personal emergency alarm system<br>▪ Hoist/lifter<br>▪ Robot vacuum<br>▪ Medical Alert system with risk of falls/duress alarm
**Chronic Obstructive Pulmonary Disease**		
Function	Basic needs	Same as Nil chronic diseases category
	Health monitoring devices/chronic disease related	▪ Heart rate monitor<br>▪ Asthma monitoring<br>▪ Spirometer<br>▪ Pulse oximeter<br>▪ Oxygen therapy<br>▪ Medication tablet reminder<br>▪ Nebuliser system<br>▪ Air purifier
	Mobility	▪ Recliner Chairs<br>▪ Electric motorised wheel chair<br>▪ Electric scooter (*assumption- wheelchair or scooter)
	Cognition	▪ Pill/Medication organises and reminder<br>▪ Personal emergency alarm system

	Environment/Home safety	▪ Fall detectors/Motion sensors/Fall monitor<br>▪ Hospital bed/electric bed<br>▪ Hoist/lifter<br>▪ Robot vacuum<br>▪ Medical Alert system with risk of falls/duress alarm
**Diabetes mellitus**		
Function	Basic needs	Same as Nil chronic diseases category
	Health monitoring devices/chronic disease related	▪ Blood glucose monitoring<br>▪ Medication tablet reminder<br>▪ Home monitoring devices for vital signs
	Mobility	▪ Recliner chairs
	Cognition	▪ Pill/Medication organises and reminder<br>▪ Personal emergency alarm system
	Environment/Home safety	▪ Fall detectors/Motion sensors/Fall monitor<br>▪ Robot vacuum<br>▪ Medical Alert system with risk of falls/duress alarm
	Exercise and Fitness	▪ Exercise bike
**Mental Health Conditions**		
Function	Basic needs	Same as Nil chronic diseases category
	Cognition	▪ Pill organises and reminder
	Environment/Home safety	▪ Global positioning system (GPS) (to locate patient if required)<br>▪ Personal emergency alarm system<br>▪ Door alarm<br>▪ Cordless bed alarm<br>▪ Medical Alert system with risk of falls/duress alarm
