# Supplementary material for: Developing a population wide cost estimating framework and methods for technological intervention enabling ageing in place: An Australian case
Source: PLoS One. 2019 Jun 26;14(6):e0218448. doi: 10.1371/journal.pone.0218448 (PMC6594731; doi:10.1371/journal.pone.0218448)
Supplement: S2 Appendix — (PDF) [file pone.0218448.s002.pdf]

S2 Appendix: List of price of digital assistive technologies for elderly people

Item	Description	Price/Price Range $AUD	Reference
**Household/Environment/Safety**			
Door alarm- Vitalcom Victoria (formerly Marker Engineering) (Boronia)	This compact, attractive door alarm is perfect for the wandering Alzheimer's patient.	89- 95	https://ilcaustralia.org.au/products/5110?search_tree=636 https://www.healthsaver.com.au/healthsaver-online-store/Anti-Wandering-Stop-Alarm-p88767070
Cordless bed alarm/ personal alarm	Alerts a caregiver when their patient gets up, up to 100' away from the patient's room. Cordless operation allows greater caregiver freedom and provides a more calm environment for the patient.		
Motion sensor	detects falls by censoring motion	130	https://www.healthsaver.com.au/healthsaver-online-store/Motion-Sensor-Fall-Alarm-p88767090
Loud power failure alarm with flashlight	Loud alarm when there is a power failure, flashlight available for senior to navigate around while there is a power outage to prevent falls.		
Medical alert system/ duress alarm/emergency pendants/ fall detection/Push button personal emergency response system	Pendant operates on the 3, 4 and 5G mobile networks. Can alert up to 5 pre-programmed contacts when the SOS button is pushed. Verbal communication is possible via the speaker and microphone in the pendant, once a contact has answered the call. The pendant also sends a text message giving GPS location via a link to Google Maps. It has a built in falls detector which automatically sends an alert.	300-497	https://ilcaustralia.org.au/products/21394?search_tree=632 https://www.healthsaver.com.au/healthsaver-online-store/Smart-Caregiver-Fall-Alarms-c24229107
AbleNet Mini Beamer Wireless Switch	A small rectangular wireless switch that is activated within 10mm of the sensor. (Actual contact with the device is not required)	192-355	
Fall Monitor	A portable wireless audible and visual alarm that provides up to 100 metres monitoring range with a variety of sensors including Smart Caregiver sensor pads and mats, Smart Caregiver motion sensors, and nurse call systems. The portable alarm can be paired with up to six wireless sensors. The device is battery operated or has an optional 12 v DC power adapter.	148-237	
	Smart Caregiver Economy Cordless Fall Monitor	148	
	Quiet Wireless Fall Alarm Monitor (Pager Not Included)	237	https://www.healthsaver.com.au/healthsaver-online-store/Quiet-Wireless-Fall-Alarm-Monitor-Pager-Not-Included-p89327296
Malem Wireless Bedwetting Alarm	A wireless enuresis alarm which uses a small magnetic transmitter attached the waistband of the user's underwear and a receiver which can be positioned up to approximately 25 metres from the transmitter. A second receiver can be purchased to be placed in the parent's room. A clip on sensor is attached to user's close fitting underwear which is connected to the transmitter. Ideal for those who prefer not to wear a pyjama top to bed.	220	
Robot Vacuum	Robotic vacuum cleaner.	589.00-1499.00	https://www.thegoodguys.com.au/vacuums-and-cleaners/vacuum-cleaners/robot-vacuums

Touch Lamp		15.00-21.89	https://www.bunnings.com.au/ http://www.kmart.com.au/category/home-&-entertainment/home-by-category/home-decor/lighting/lamps/251205
Lamp switch turners	Not digital; helps when turning on lamps		
Adaptive switches		80-129	https://ilcaustralia.org.au/products/5534
Automatic door opener	for those with mobility issues		
Talking thermostat	annouces day, date, time and temperature		
**ICT**			
Mobile devices i.e. phones, iPad, smart watches	medical consultation wth specialists		
ipad		439-1919	https://www.harveynorman.com.au/catalogsearch/result/index/?dir=asc&order=price&q=ipad+#toolbar-top
TV (Toshiba 32-inch HD LED LCD Smart TV)	video link appointment	395	
Computer		298-1698	https://www.harveynorman.com.au/catalogsearch/result/?af=producttype%3Alaptops&q=laptop+computer
**Vision & Hearing Products**			
Talking Rx	Seniors with poor vision can take their medications correctly with the Talking Rx's talking directions.	24.95	https://www.afb.org/prodProfile.asp?ProdID=799
Telephone amplifier/door bell and telephone ring amplifier	Portable phone amplifier; amplifies sounds- rings and flash	79.95- 199	https://ilcaustralia.org.au/products/21490 https://ilcaustralia.org.au/products/17129
Sound amplifier	Pocketalker Ultra System amplifies sounds closest to the listener while reducing background noise	225-603.95	https://ilcaustralia.org.au/ https://www.aldsaustralia.com.au/categories/personal-sound-amplifiers/page/3/
Big button phone	This picture phone is absolutely fantastic for those that have sight issues.	129	https://ilcaustralia.org.au/products/13918
Audio books	vision impairment		
Amplified phone		182.95-237.95	https://www.aldsaustralia.com.au/categories/personal-sound-amplifiers/
Pocket talker	Personal sound amplifier	284.95-338.95	https://www.aldsaustralia.com.au/categories/personal-sound-amplifiers/
Hearing Aids	Costing obtained from internet	3340-14000	https://www.choice.com.au/health-and-body/optical-and-hearing/hearing/buying-guides/hearing-aids
**Exercise & Fitness Products**			
Power Plate Exercise Machine	Uses whole body vibration and acceleration training to help heal injuries and provide a low impact workout.	-	
Pedal exercisers/pedal bike	Fully automatic electronic exercise pedal	165-278.30	https://ilcaustralia.org.au/products/11026

Treadmill		995-2999.00	http://search.rebelsport.com.au/search?isort=price&lbc=rebelsport&method=and&p=Q&ts=custom&uid=106041558&w=treadmill&cnt=100
Exercise bike		149.99-1999.99	http://search.rebelsport.com.au/search?p=Q&srid=S1-2SYDP&lbc=rebelsport&ts=custom&w=exercise%20bikes&uid=106041558&method=and&isort=price&view=grid&srt=12
**Bedroom & Sleeping Products**			
Hospital bed/electric bed	Offers the greatest convenience for the patient and caregiver.	1890-2400	http://at-aust.org/items/13724
Hoist/lifter		3275	
Nursing bed/ Princess bed		2500	
**Health & Medical Supplies Products**			
Portable oxygen	You can now take off on a moment's notice, without having to watch the clock or guess how long your oxygen will last. Oxygen travel made, easy-airline approved. [$89 to start up + $46 monthly hire + $39 oxygen (price for 3 bottle 2L each bottle last for 3 hours -->depends on what the prescription is)]	*$174	http://www.heesonmedical.com.au/?page_id=66<br>07 3262 5506
Blood glucose meter/ conitnuous glucose monitoring	Range. Test blood glucose levels	$29-65.25	http://www.vitalmed.com.au/images/pdf/catalogue/2018_19/vms_diagnostic-reagents.pdf
	Accu chek	65.25	https://store.independenceaustralia.com/accu-chek-blood-glucose-meter.html
	Accuchek Performa Blood Glucose Meter - ROC51551	45	http://zonemedical.com.au/medical-equipment/Blood-Monitoring/Accuchek-Performa-Blood-Glucose-Meter-ROC51551_2.html
Medication tablet reminder	Alarm to remind patients to take their tablets [TabTimer Pill Box Reminder TT4-3]	51.04-79.75	
INR measurement tester	Alere INRation 2 PATIENT/INR Monitoring system	595	
Blood pressure monitor/sphygmomanometer	Range. Test blood pressure	49- 749	http://zonemedical.com.au/medical-equipment/Blood-Pressure-Monitors/Omron-HEM907-Digital-Blood-Pressure-Monitor-JADHEM907_2.html
	Omron HEM907 Digital Blood Pressure Monitor - JADHEM907		
Blood pressure monitor/sphygmomanometer	HEM7121 Standard Automatic Blood Pressure Monitor	$115	
	Honsun LD578 Automatic Digital Blood Pressure Monitor - HONLD578	$49	http://zonemedical.com.au/medical-equipment/Blood-Pressure-Monitors/Honsun-LD578-Automatic-Digital-Blood-Pressure-Monitor-HONLD578.html
Electronic Asthma Monitoring	electronic peak flow meter	$149	-

Hand held device to measure cholesterol, glucose, lactate and triglycerides<br>Accutrend Plus		$295	http://www.vitalmed.com.au/images/pdf/catalogue/2018_19/vms_diagnostic-reagents.pdf
Thermometer	Range	9.45-59.62	
	Monitor temperature	$9.45	
	Tympanic with covers	$59.62	https://store.independenceaustralia.com/thermometer-tympanic-with-covers.html
Air mattress	To prevent pressure injuries	1100-4700	https://ilcaustralia.org.au/
Carilex Dual Alternating Pressure Mattress Replacement		4996	http://at-aust.org/items/10248
Nebuliser System - 2.4MHz Ultrasonic Nebulizer		45	https://www.caremax.com.au/ultrasonic-nebulizer.html?gclid=EAIaIQobChMIv6Xi7pGW2wIVRB0rCh1jnAGREAYYASABEgJ8rvD_BwE
	Machine used to change liquid medication into vapour for inhalation	109-225	http://www.vitalmed.com.au/images/pdf/catalogue/2018_19/vms_diagnostic-reagents.pdf
Nebuliser System	purify air for allergy sufferers		
Air purifier	i.e. diabetes mellitus, cardiovascular disease, cardiac arrhythmias, chronic obstructive pulmonary disease, Chronic Kidney Disease		
Home monitoring devices/ vital signs	alert pts regarding unsafe dose		
Drug delivery/infusion pump	remote monitoring of continence events using a sensor in a disposable continence aid. Helps to aid personalised are plan		
Smart incontinence system	Monitor heart rate	79.95-699	http://www.rebelsport.com.au/store/gym-fitness/heart-rate-monitors/412?pageSize=12&sort=ProductVisiblePrice&page=1
Heart rate monitor	diagnostic and assessment tool that measures and records the electrical activity of the heart using electrodes attached to the skin	79.95	https://omronhealthcare.com.au/product-category/omron-heart-rate-monitors/
Electrocardiogram (ECG)	Self monitoring ECG- The Heart Check Pen Handheld ECG	USD $259	http://www.theheartcheck.com/wheretobuy_ECGPEN.html
	monitoring sytem for patients with kidney disease undergoing home haemodialysis		
Home haemodialysis monitor	Measure volume of air inspired and expired by the lungs	99-2495	http://zonemedical.com.au/medical-equipment/spirometers/MIR-Smart-One-Personal-Spirometer-Peak-Flow-Meter-MIR9111000D2.html
Spirometer	Measuring the pulse oxygen saturation and pulse rate through finger	49-1795	http://zonemedical.com.au/medical-equipment/pulse-oximeters/

Pulse Oximeter	Pulse Oximeter	622.93	https://store.independenceaustralia.com/pulse-oximeter-2.html
	Fingertip pulse oximeter	129.09	https://store.independenceaustralia.com/fingertip-pulse-oximeter.html
	Back Belt with Magnetic and Heating Therapy. It uses 2 magnets and USB powered heating pad to warm up strained tendons, muscles and bones to relieve back pain, soreness, tightness and discomfort.	81.99-97.99	https://www.caremax.com.au/pain-relief/supports-braces/pain-management-braces-belts.html
Thermal belt	Transcutaneous electrical nerve stimulation- used for pain relief	116-198	
TENS	Transcutaneous electrical nerve stimulation- used for pain relief	116-198	
**Mobility**			
Motorised wheel chair	Heartway P3D Maxx 20	1790	
	Range	1699.99-6125.99	
	Jazzy Air Wheelchairs electric	6125.99	
	Cobalt Travel Power Wheelchair	1699.99	
Home elevators	Range	1099.99-10409.99	
Electric Mobility Scooter	Predator 4-wheel drive mobility scooter	10409.99	http://ilsau.com.au/department/mobilityscooters/?gclid=EAIaIQobChMIk-z1n9KW2wIVkB0rCh14fAQREAAYASACEgKr5fD_BwE
	Easy Rider Mobility Scooter	5555.99	
	Scout Portable Economy Mobility Scooter	1099.99	
	Pride Go-Go LX 3 wheel with CTS suspension	1564	
Stair lifts	Range	795.99-3800.99	
Life chair/ recliners	Assist to help sit down and stand up	1455	
	Pride C5 Electric recliner lift chair	895.99	
	K Care Compact Electric Recliner Lift Chair	3800.99	
	Mayfair Select Electric Lift chair	795.99	
**Organisation tools**			
Walking cueing device	Clips onto a belt or waistband. It continuously monitors the user's walking motion with a small motion sensor and intelligent software.	2199	http://at-aust.org/items/10247
